# Facilitator Contact, Discussion Boards, and Virtual Badges as Adherence Enhancements to a Web-Based, Self-guided, Positive Psychological Intervention for Depression: Randomized Controlled Trial

**DOI:** 10.2196/25922

**Published:** 2021-09-22

**Authors:** Judith Tedlie Moskowitz, Elizabeth L Addington, Eva Shiu, Sarah M Bassett, Stephanie Schuette, Ian Kwok, Melanie E Freedman, Yan Leykin, Laura R Saslow, Michael A Cohn, Elaine O Cheung

**Affiliations:** 1 Department of Medical Social Sciences Osher Center for Integrative Medicine Northwestern University Feinberg School of Medicine Chicago, IL United States; 2 Department of Obstetrics and Gynecology Biological Sciences Division University of Chicago Chicago, IL United States; 3 Department of Psychology and Neuroscience Duke University Durham, NC United States; 4 Department of Psychiatry and Behavioral Sciences Osher Center for Integrative Medicine Northwestern University Feinberg School of Medicine Chicago, IL United States; 5 Department of Psychology Palo Alto University Palo Alto, CA United States; 6 Department of Health Behavior and Biological Sciences University of Michigan School of Nursing Ann Arbor, MI United States; 7 Osher Center for Integrative Medicine University of California San Francisco San Francisco, CA United States

**Keywords:** mHealth, adherence, depression, discussion board, gamification, positive psychological intervention, mobile phone

## Abstract

**Background:**

Adherence to self-guided interventions tends to be very low, especially in people with depression. Prior studies have demonstrated that enhancements may increase adherence, but little is known about the efficacy of various enhancements in comparison to, or in combination with, one another.

**Objective:**

The aim of our study is to test whether 3 enhancements—facilitator contact (FC), an online discussion board, and virtual badges (VB)—alone, or in combination, improve adherence to a self-guided, web-based intervention for depression. We also examined whether age, gender, race, ethnicity, comfort with technology, or baseline depression predicted adherence or moderated the effects that each enhancement had on adherence.

**Methods:**

Participants were recruited through web-based sources and, after completing at least 4 out of 7 daily emotion reports, were sequentially assigned to 1 of 9 conditions—the intervention alone; the intervention plus 1, 2, or all 3 enhancements; or an emotion reporting control condition. The intervention was a positive psychological program consisting of 8 skills that specifically targeted positive emotions, and it was delivered over 5 weeks in a self-guided, web-based format. We operationalized adherence as the number of skills accessed.

**Results:**

A total of 602 participants were enrolled in this study. Participants accessed, on average, 5.61 (SD 2.76) of 8 skills. The total number of enhancements participants received (0-3) did not predict the number of skills accessed. Participants who were assigned to the VB+FC condition accessed significantly more skills than those in the intervention only conditions. Furthermore, participants in arms that received the combination of both the VB and FC enhancements (VB+FC and VB+FC+online discussion board) accessed a greater number of skills relative to the number of skills accessed by participants who received either VB or FC without the other. Moderation analyses revealed that the receipt of VB (vs no VB) predicted higher adherence among participants with moderately severe depression at baseline.

**Conclusions:**

The results suggested that the VB+FC combination significantly increased the number of skills accessed in a self-guided, web-based intervention for elevated depression. We have provided suggestions for refinements to these enhancements, which may further improve adherence.

**Trial Registration:**

ClinicalTrials.gov NCT02861755; http://clinicaltrials.gov/ct2/show/NCT02861755

## Introduction

Despite the fact that up to 20% of people in the United States will experience depression over the course of their lives [1], more than half will remain undiagnosed and untreated [2] for a variety of reasons including treatment cost and geographic inaccessibility [3,4]. Web-based self-help or self-guided (eHealth) programs can overcome potential barriers associated with conventional modes of treatment and may be particularly useful for individuals who might not otherwise seek out conventional face-to-face therapy [5]. However, eHealth interventions for depression also come with significant challenges. Retention in these web-based, self-guided interventions is low compared with other types of trials [6]. For example, previous studies reported retention rates as low as 43% posttreatment, with even lower rates with longer-term follow-up [7]. A recent review of retention in studies of smartphone-delivered interventions for mental health concluded that researchers can expect attrition of up to one-third of participants who enroll in the study [8]. In addition, adherence to intervention content—operationalized as the number of log-ins, duration of web exposure, or number of modules or exercises completed—is as low as 50% for web-based programs [7], and complete adherence to all components of smartphone-delivered interventions for depression was achieved by an average of only 34% of participants [8]. Predictors of poor adherence in web-based depression trials include higher baseline levels of depression [7], male gender [9,10], and lower levels of education [9]. Older age is sometimes found to be associated with better adherence [9,10] and sometimes poorer adherence [7]. Those with less frequent internet use at baseline (weekly to monthly compared with daily) may be more adherent to self-guided programs [10].

Social enhancements such as discussion boards that encourage users to generate and share content with each other can improve engagement [11-14]. Opportunities to earn points and badges can also increase motivation by providing virtual rewards to participants in the form of positive feedback [15-17]. More conventional enhancements, such as reminders via postcards or brief phone calls, can also increase participation [18,19], although other studies have found that similar enhancements such as personalization, interactivity, reminders, and text messages were not associated with better intervention adherence [20]. The discrepancy in these results suggests that specific automated enhancements, or combinations of automated and human-supported enhancements, may be necessary to maximize adherence to self-guided programs.

In this study, we tested the following three enhancements to a self-guided web-based intervention for people experiencing depressive symptoms: brief facilitator contact (FC), an online discussion board (ODB), and virtual badges (VB). These features were developed in previous phases of the program [21,22] and were selected based on research suggesting that personal contact, interactive components, and incentivizing participation increased engagement in web-based programs [7,23-29].

One enhancement was brief facilitator phone contact. Contact from study staff, a mental health professional, or another type of coach can provide a sense of obligation or accountability on the part of the participant and increase adherence to intervention content [29]. In eHealth interventions, this social presence, or sense that there is another human behind the intervention who is aware of the participant’s engagement and may provide reminders or prompts, significantly improves adherence [30,31]. FC may increase user engagement by building rapport and connection to the intervention, providing feedback, or supporting meaningful use by tailoring the content or application of the material to participants’ needs [32].

The second enhancement we tested was an ODB. Asynchronous discussion boards where participants can reflect on their experiences of learning the skills presented in the intervention and interact with other participants may provide a sense of peer support that improves the efficacy of the intervention. Although web-based support groups alone have not been particularly effective [33], from a collaborative learning perspective, the addition of a forum that allows participants to interact asynchronously with other participants and intervention content may help facilitate understanding and retention of the content and increase engagement with the targeted behaviors [34].

VB are a common form of gamification that increases engagement with an intervention [35] through the inclusion of playful, enjoyable, or competitive elements that boost adherence to a designated activity [36]. Although VB have been criticized for their emphasis on extrinsic motivation at the expense of intrinsic [35], there is evidence that gamified interventions are more engaging than those that do not contain such elements [37].

We hypothesize that the enhancements would increase adherence, operationalized as the number of sessions accessed, and aim to explore whether the combination of enhancements had a significantly greater impact than any one alone. We also examine whether age, gender, race or ethnicity, comfort with technology, or baseline depression predict adherence or moderate the effects of each enhancement on adherence. Finally, we explore enjoyment ratings as predictors of use or engagement with each enhancement.

## Methods

### Participants

Detailed methods are described in the study by Cheung et al [22]. The study was approved by the institutional review board and preregistered through ClinicalTrials.gov (trial number: NCT02861755). All participants were recruited on the web through platforms such as ResearchMatch, Craigslist, and Reddit. Eligibility was determined using a web-based screener. To be eligible for the study, participants had to (1) have at least mild levels of depression, as indicated by a Patient Health Questionnaire-8 (PHQ-8) depression score of ≥5 [38]; (2) be aged ≥18 years; (3) have daily access to the internet; (4) own a mobile phone; (5) live in the United States; and (6) be able to read and write in English.

### Procedures

#### Baseline and Randomization

After obtaining web-based consent, participants completed a baseline questionnaire and a 7-day run-in period in which they were asked to complete brief daily assessments of emotion. If participants completed the baseline assessment and at least 4 of 7 days of the run-in period, they were allocated to one of the nine study arms. We stratified participant allocation to a condition based on gender and the level of depressive symptom severity (PHQ-8 score: 5-9 [mild]; 10-14 [moderate]; 15-19 [moderately severe]; >20 [severe]) to ensure sufficient numbers of each group within each condition, and participants were sequentially assigned to the study arm after stratification.

The nine study arms varied according to the type and number of adherence enhancements. The nine conditions are as follows: (1) intervention alone, (2) intervention+FC, (3) intervention+ODB, (4) intervention+VB, (5) intervention+FC+ODB, (6) intervention+FC+VB, (7) intervention+ODB+VB, (8) intervention+FC+ODB+VB, and (9) the emotion reporting control condition.

#### Enhancements

In the FC conditions, the study staff called participants once per week to check their progress through the course. Specifically, following a prescribed set of questions, the staff member asked about the participant’s experience learning that week’s skills, using the home practice, and completing the daily emotion surveys; they probed for questions about the skills, accomplishments, barriers, and any difficulties with the technology. If the participant could not be reached by phone, the staff member sent an email with the same content in place of the call and encouraged the participant to reply if they had questions. Staff members were explicitly trained not to provide supportive counseling or skill training, but instead focused on discussing progress, challenges, and technology issues.

Participants who were assigned to the ODB had access to a virtual discussion board where they could participate anonymously using pseudonyms. The study staff provided prompts to seed the discussion board and were notified when a participant posted something. Posts were reviewed for inappropriate content and signs of extreme distress or suicidality.

In the VB condition, participants could earn flower badges that they could arrange in a garden plot. They received badges for completing activities such as creating a profile, reading a lesson, or logging in to the website for 7 consecutive days.

#### MARIGOLD Intervention

Conditions 1-8 received the MARIGOLD program, a positive psychological intervention that consisted of eight skills that specifically targeted positive emotions: noticing positive events, capitalizing, gratitude, behavioral activation, mindfulness, positive reappraisal, personal strengths, and acts of kindness. Additional details of the positive emotion skills intervention can be found elsewhere [21,22,39-43]. The skills were delivered over the course of 5 weeks in a self-guided, web-based format, and each week had associated home practice activities. Participants did not have access to all the skills at once; instead, 1 to 3 skills were released in each of the 5 weeks. Participants in conditions 1 to 8 also completed the same daily emotion reporting as the control condition (described in the following section).

#### Emotion Reporting Control

Participants in arm 9 were asked to log in to the website once per day for 49 days (the length of the other eight conditions) to report their emotions. Participants in the emotion reporting control arm completed approximately 20 of the 49 days of daily emotion reporting during the study period (mean 19.90, SD 18.01; range 0-50). They did not receive any of the intervention content and thus did not have adherence data as we have defined it; participants in the control condition will therefore not be included in the present analyses (we have included greater detail on the rates of retention for participants in the emotion reporting control arm and the comparison of daily emotion reporting in the emotion reporting control arm versus the other eight conditions in the supplemental analyses; Multimedia Appendix 1).

### Measures

#### Adherence and Enhancement Ratings

The primary adherence outcome was operationalized as the number of skills (out of eight) accessed (see supplemental analyses in Multimedia Appendix 1 for analyses with other operationalizations of adherence [number of pages viewed and home practice completed] and study retention. Predictors of alternative operationalizations of adherence and of retention were essentially the same). In addition, we explored the evaluations of the enhancements, measured both quantitatively and qualitatively. Upon completion of the 5-week MARIGOLD study, participants completed postcourse surveys in which they provided feedback about the intervention content and enhancements. Participants were asked to rate whether they enjoyed each enhancement they were assigned to receive (ie, FC, ODB, and VB) on a slider scale from 0 (*definitely not*) to 100 (*definitely yes*). Participants also asked open-ended questions regarding the benefits, drawbacks, and impact of each of the enhancements they received.

#### Predictors of Adherence and Enhancement Ratings

We also examined demographics, baseline depression, and comfort with technology as predictors of adherence and enjoyment of enhancements. Gender was assessed as *male*, *female*, *or other*, or *prefer not to answer*. Participants were asked whether they considered themselves Hispanic or Latino or Latina and how they self-identified their race (*Black*, *White*, *Asian*, *Native American*, *Pacific Islander*, and *mixed or other*). Age was calculated based on the date of birth. Depression was assessed using the PHQ-8 [38]. Finally, comfort with technology was assessed with six items that tapped a participant’s overall frequency of and confidence with technology use (eg, “I am confident I can navigate websites”) with response options ranging from 0 (*not at all confident*) to 10 (*totally confident*).

### Analyses

#### Baseline Predictors of Adherence and Engagement With Enhancements

We first conducted an overdispersed (or quasi-) Poisson regression [44,45] to examine whether the number of enhancements received predicted the number of skills accessed. We then conducted the analyses predicting the number of skills accessed from the intervention arm, with the intervention only condition as the reference category.

We then conducted overdispersed Poisson regressions predicting the number of skills accessed from age, gender (1=female; male=0), race or ethnicity (with indicator variables to represent each race or ethnicity category), comfort with technology, and baseline depressive symptom severity (categorized as mild, moderate, moderately severe, and severe).

To further examine whether adherence to the study differed as a function of the combination of enhancements received, we conducted an additional overdispersed Poisson regression predicting each adherence outcome as a function of whether the participant received each enhancement (FC, ODB, and VB), the FCODB, FCVB, and ODBVB two-way interactions and the FCODBVB three-way interaction. The two-way and three-way interactions allowed us to explore whether receiving a specific combination of enhancements had a significantly greater impact than receiving any one enhancement alone.

#### Moderators of Adherence

We examined whether the effect of each enhancement on adherence was moderated by the following characteristics: age, gender (female vs male), ethnicity (Hispanic vs non-Hispanic), race (Asian, Black or African American, White, other race [Pacific Islander, Native American, and mixed or other]), comfort with technology, and baseline depressive symptom severity (mild, moderate, moderately severe, and severe). To examine this, overdispersed Poisson regressions predict adherence by whether the participants received each enhancement, with dummy variables representing each enhancement, the moderator of interest, and the two-way interactions between the moderator and each enhancement type (FC×moderator, ODB×moderator, and VB×moderator). Owing to power limitations, we did not test all 4 moderators in the same model or explored interactions among the moderators and combinations of enhancements.

#### Power Analysis

A sensitivity power analysis using the G*Power 3.1.9.6 software [46] revealed that the sample size for this study (N=539 participants across the eight intervention arms) was sufficient to detect a small effect size with adequate power. The minimum effect size necessary for this study was *w*=0.12, assuming an α significance criterion of *P=.*05 and power=0.80.

## Results

### Screening, Enrollment, and Randomization

**Figure 1:**
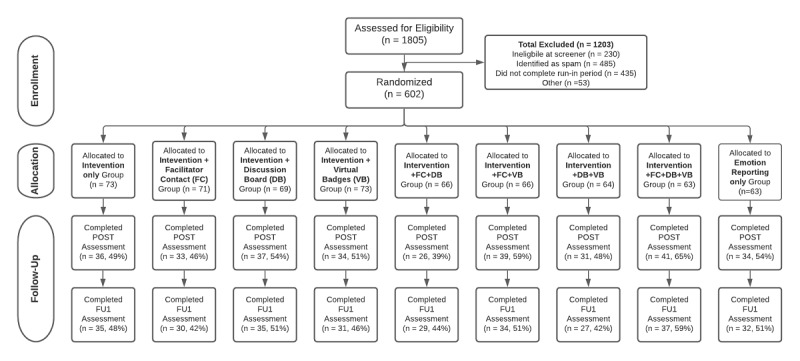
CONSORT (Consolidated Standards of Reporting Trials) diagram. FU: follow-up.

Of the 1805 respondents who completed the screener, 1037 (57.45%) were deemed eligible and entered the run-in period. Of these, 58.05% (602/1037) completed at least 4 of 7 days of the emotion reports for the run-in and were sequentially assigned to one of the nine study conditions. Figure 1 shows the CONSORT (Consolidated Standards of Reporting Trials) diagram. There were 485 eligibility forms that were deemed spam. Most of these cases were identified because they enrolled in quick succession on the same day and had suspicious repeating patterns of first or last names. We sent additional screener questions to these cases and none completed them and therefore did not proceed to enrollment.

### Baseline Characteristics

Across the nine conditions, there were no differences in baseline demographics, depression, or comfort with technology (Table 1). The average age of the participants was 38 years (range 18-80 years). Three-fourths of the sample were female (422/573, 73.6%), three-fourths were White (421/573, 73.4%), and 43% (244/568) were college graduates. Compared with those who did not complete at least 4 of the 7 days of the run-in period, participants who completed the run-in were older (mean 37.95, SD 13.68 vs mean 36.03, SD 12.77; *P*=.04), more likely to be female (467/633, 73.7% vs 210/323, 65%; *P=.*008), and had lower baseline depressive symptom severity (PHQ-8: mean 13.85, SD 5.18 vs mean 15.34, SD 5.11); *P*<.001; Table S1 in Multimedia Appendix 1).

**Table 1:** Sample demographics and baseline variables by condition.

Baseline variables		Total sample (N=602)	Condition										*P* value^a^	
			Intervention alone (n=73)	FC^b^ (n=71)	ODB^c^ (n=69)	VB^d^ (n=67)	FC+ODB (n=66)	FC+VB (n=66)	ODB+VB (n=64)	FC+ODB +VB (n=63)	Control (n=63)			
Age (years), mean (SD)		37.8 (13.5)	39.1 (13.4)	37.8 (14.5)	38.9 (14.1)	38.4 (15.5)	38.0 (12.6)	38.4 (14.2)	36.1 (12.1)	38.6 (12.8)	34.7 (12.1)	.67		
**Gender, n (%)**														.85
	Male	146 (26)	18 (25)	17 (25)	18 (26)	16 (25)	16 (25)	17 (27)	15 (25)	13 (25)	16 (25)			
	Female	422 (74)	51 (72)	48 (72)	49 (72)	49 (75)	47 (75)	46 (73)	46 (75)	39 (75)	47 (75)			
**Baseline PHQ-8^e^, n (%)**														.99
	Mild	139 (23)	17 (23)	16 (23)	16 (23)	16 (24)	16 (24)	16 (24)	14 (22)	14 (22)	14 (22)			
	Moderate	193 (32)	23 (32)	23 (32)	21 (30)	21 (31)	21 (32)	21 (32)	21 (33)	21 (33)	21 (33)			
	Moderately severe	175 (29)	21 (29)	21 (30)	21 (30)	19 (28)	19 (29)	19 (29)	19 (30)	18 (29)	18 (29)			
	Severe	95 (16)	12 (16)	11 (16)	11 (16)	11 (16)	10 (15)	10 (15)	10 (16)	10 (16)	10 (16)			
**Ethnicity, n (%)**														.89
	Hispanic	100 (17)	13 (18)	8 (12)	11 (16)	10 (15)	13 (21)	11 (18)	12 (20)	12 (23)	10 (16)			
	Non-Hispanic	473 (83)	58 (82)	59 (88)	57 (84)	55 (85)	50 (79)	52 (83)	49 (80)	40 (77)	53 (84)			
**Race, n (%)**														
	Black	93 (16)	8 (11)	7 (10)	15 (22)	9 (14)	13 (21)	12 (19)	10 (16)	9 (17)	10 (16)	.62		
	White	421 (74)	(73)	(73)	(74)	(79)	(68)	(73)	(67)	(79)	(75)	.86		
	Asian	49 (9)	9 (13)	11 (16)	4 (6)	4 (6)	4 (6)	6 (10)	5 (8)	3 (6)	3 (5)	.26		
	Native American	27 (5)	2 (3)	5 (8)	4 (6)	4 (6)	3 (5)	4 (6)	2 (3)	0 (0)	3 (5)	.71		
	Pacific Islander	2 (1)	0 (0)	0 (0)	1 (2)	0 (0)	0 (0)	1 (2)	0 (0)	0 (0)	0 (0)	.56		
	Mixed or other	43 (8)	4 (6)	5 (8)	5 (7)	7 (11)	5 (8)	2 (3)	8 (13)	1 (2)	6 (10)	.38		
**Education, n (%)**														.33
	<High school	4 (1)	0 (0)	0 (0)	1 (2)	0 (0)	2 (3)	0 (0)	1 (2)	0 (0)	0 (0)			
	High school	40 (7)	4 (6)	2 (3)	10 (15)	6 (9)	5 (8)	7 (11)	2 (3)	2 (4)	2 (3)			
	Some college	170 (30)	23 (32)	21 (32)	15 (22)	23 (35)	16 (26)	18 (29)	23 (38)	16 (31)	15 (25)			
	College graduate	244 (43)	23 (32)	34 (52)	32 (47)	26 (40)	27 (44)	28 (44)	21 (34)	21 (40)	32 (53)			
	Professional degree	98 (17)	18 (25)	8 (12)	9 (13)	9 (14)	12 (19)	8 (13)	12 (20)	12 (23)	10 (17)			
	Other	12 (2)	3 (4)	1 (2)	1 (2)	1 (2)	0 (0)	2 (3)	2 (3)	1 (2)	1 (2)			
**Income (US $), n (%)**														.24
	<10,000	48 (8)	10 (14)	4 (6)	8 (12)	3 (5)	8 (13)	6 (10)	5 (8)	2 (4)	2 (3)			
	10,000 to 19,999	65 (11.5)	11 (16)	6 (9)	9 (13)	11 (17)	10 (16)	4 (7)	4 (7)	4 (8)	6 (10)			
	20,000 to 29,999	92 (16)	11 (15)	10 (15)	13 (19)	12 (19)	11 (18)	8 (13)	7 (12)	10 (19)	10 (15)			
	30,000 to 49,999	102 (18)	10 (14)	15 (23)	16 (24)	13 (20)	9 (15)	10 (16)	10 (16)	10 (19)	9 (15)			
	50,000 to 74,999	105 (19)	10 (14)	13 (20)	11 (16)	10(15)	7 (11)	13 (21)	9 (15)	13 (25)	19 (31)			
	75,000 to 99,999	72 (13)	10 (14)	6 (9)	5 (8)	7 (11)	7 (11)	10 (16)	17 (28)	3 (6)	7 (12)			
	100,000 to 199,999	68 (12)	7 (10)	10 (15)	4 (6)	9 (14)	7 (11)	8 (13)	9 (15)	7 (14)	7 (12)			
	>200,000	14 (2)	1 (1)	2 (3)	1 (2)	0 (0)	3 (5)	3 (5)	0 (0)	3 (6)	1 (2)			
Comfort with technology, mean (SD)		9.2 (1.3)	9.1 (1.6)	9.3 (1.2)	9.2 (1.2)	9.1 (1.5)	9.2 (1.2)	9.0 (1.3)	9.2 (1.3)	9.1 (1.7)	9.5 (0.7)	.57		

^a^*P* values were based on analysis of variance (continuous) and chi-square tests (categorical outcomes).

^b^FC: facilitator contact.

^c^ODB: online discussion board.

^d^VB: virtual badges.

^e^PHQ-8: Patient Health Questionnaire-8.

### Adherence

**Figure 2:**
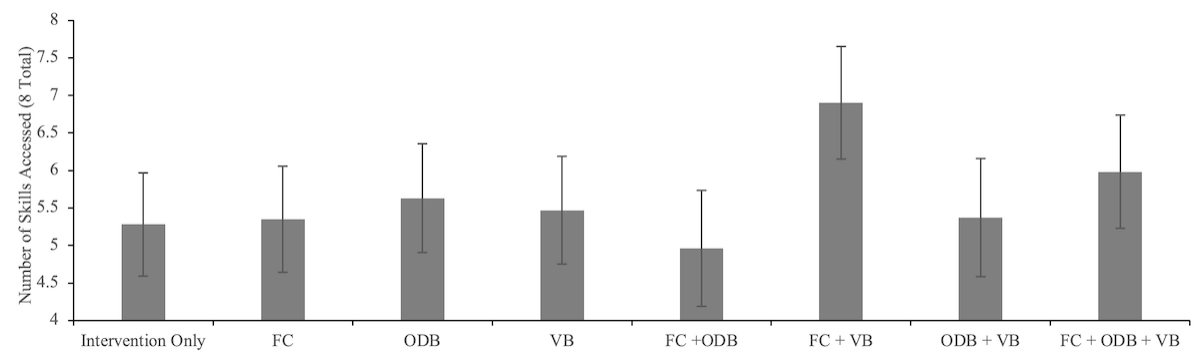
Number of skills accessed (n=8) as a function of condition. Error bars denote 95% CIs. FC: facilitator contact; ODB: online discussion board; VB: virtual badges.

Participants accessed, on average, 5.61 (SD 2.76) of 8 skills. The total number of enhancements that participants received (0-3) did not predict the number of skills accessed (1 enhancement vs 0: *P=*.63; 2 enhancements vs 0: *P*=.25; 3 enhancements vs 0: *P*=.19). We conducted an analysis predicting the number of skills accessed as a function of whether the participant received each enhancement and their two- and three-way interactions. There were no significant main effects for the FC (*P*=.24) or ODB (*P*=.36) enhancements. There was a significant main effect of receiving the VB enhancement, such that participants in the conditions that received VB (VB, VB+FC, VB+ODB, and VB+FC+ODB) had a greater number of skills (mean 5.90, 95% CI 5.52-6.31) relative to participants in groups that did not receive the VB enhancement (intervention only, FC, ODB, and FC+ODB; mean 5.30; range 4.96-5.67; χ^2^_1_=5.0; *P=.*03). This main effect, however, appeared to be driven by a significant VB by FC two-way interaction predicting the number of skills accessed (χ^2^_1_=5.5; *P=.*02) such that participants in arms that received the *combination* of both the VB and FC enhancements together (VB+FC and VB+FC+ODB) accessed a greater number of skills (mean 6.42; range 5.87-7.03) than participants who received either enhancement without the other (VB without FC: mean 5.42; range 4.92-5.98; *P*=.01; FC without VB: mean 5.15; range 4.67-5.68; *P*=.001). Furthermore, participants who received either VB or FC enhancement without the other did not differ in the number of skills accessed compared with participants who received neither enhancement (neither VB or FC: mean 5.45; range 4.98-5.98; *P*=.40 to *P*=.93). No other two-way or three-way interactions emerged with statistical significance (FC by ODB interaction: *P*=.17; ODB by VB interaction: *P*=.44; and FC by ODB by VB interaction: *P*=.94). Taken together, these findings suggest that receiving the VB and FC enhancements *in combination* increased the number of skills accessed, but receiving either the VB or FC enhancement without the other did not (Figure 2).

The patterns of results were the same for other indicators of adherence (eg, pages viewed or home practice completed) and retention in the study. The details of these analyses are provided in Multimedia Appendix 1.

### Moderators of Adherence

We examined age, gender, race, ethnicity, comfort with technology, and baseline depression as predictors of adherence and as moderators of the effects of each individual enhancement on adherence. As seen in Table 2, none of these baseline characteristics significantly predicted participants’ adherence to the intervention content (*P*=.19 to *P*=.87).

**Table 2:** Overdispersed Poisson regression predicting adherence from baseline predictors.

Predictor		Number of skills accessed (n=8)	
		Odds ratio (95% CI)	*P* value
Age (years)		1.00 (0.99-1.00)	.79
**Gender**			
	Female	1.08 (0.96-1.21)	.20
	Male (reference)	—^a^	—
**Ethnicity**			
	Hispanic	1.04 (0.91-1.19)	.60
	Non-Hispanic (reference)	—	—
**Race**			
	Asian	0.94 (0.78-1.13)	.50
	Black or African American	1.09 (0.96-1.23)	.19
	Other race^b^	0.97 (0.82-1.15)	.70
	White (reference)	—	—
Comfort with technology		1.01 (0.97-1.04)	.78
**Baseline PHQ-8^c^**			
	Moderate	1.05 (0.92-1.19)	.47
	Moderately severe	0.99 (0.86-1.13)	.87
	Severe	1.04 (0.90-1.22)	.58
	Mild (reference)	—	—

^a^Not available (indicates reference value).

^b^Other race: the other race category constitutes those who identified as Pacific Islander, Native American, mixed, or other.

^c^PHQ-8: Patient Health Questionnaire-8.

Next, we explored whether the enhancements had a greater effect on adherence depending on demographics, comfort with technology, or baseline depression. There was a significant baseline depressive symptom severity by VB two-way interaction in predicting the number of skills accessed, χ^2^_3_=8.6 (*P*=.04). Specifically, among participants with moderately severe depression (scores between 15 and 19 on the PHQ-8), receiving VB enhancement was associated with better adherence compared with those who did not receiving VB enhancement (B=1.09; *P=.*01). Two trends in the data that did not reach conventional levels of statistical significance are worth noting: for those in the mild (5-9) and moderate (10-14) range in the PHQ, there was also a positive association between the VB group and adherence (mild: B=1.00; *P=.*06; moderate: B=0.80; *P=.*10). At the highest level of depressive symptoms (scores >20); however, the association was in the opposite direction, with those who received the VB enhancement showing *lower* adherence compared with those who did not receive the VB enhancement (B=−1.21; *P=.*08). Given that the effects among participants in 3 of the 4 depression groups (mild, moderate, and severe but not moderately severe) did not reach statistical significance, caution is warranted in drawing conclusions based on these data.

### Exploratory Analyses on Engagement With Enhancements

To better understand the responses to the three enhancements, we further explored the level of engagement and enjoyment ratings for each one.

#### Analysis of FC

For FC (n=266), study staff were able to contact 45.5% (121/266) of the participants in the FC enhancement conditions by phone at least once, and the average number of weeks that staff were able to reach participants by phone was 1 (SD 1.42; range 0-6). Facilitators were able to reach participants who were older, female, and more comfortable with technology and those who had baseline depression scores in the moderate and moderately severe range more frequently than those who were younger, male, less comfortable with technology, and had baseline depression scores in the mild range (*P*=.001 to *P*=.04; Table 3). The number of weeks that participants could be reached by phone was positively correlated with the number of skills accessed (Spearman *r*_206_=0.32; *P*<.001).

**Table 3:** Overdispersed Poisson regressions predicting engagement with the virtual badges, facilitator contact, and ODB^a^ enhancements from baseline predictors.

Predictor			Number of virtual badges				Facilitator contact phone contact					ODB posts		
			OR^b^ (95% CI)		*P* value		OR (95% CI)		*P* value		OR (95% CI)			*P* value
Age (years)			1.01 (1.00-1.02)		.05		1.06 (1.03-1.08)		<.001		1.01 (0.99-1.02)			.29
**Gender**														
	Female	1.23 (0.88-1.71)		.24		4.08 (1.44-11.58)		.008		0.61 (0.40-0.92)			.02	
	Male (reference)	—^c^		—		—		—		—			—	
**Ethnicity**														
	Hispanic	1.60 (1.10-2.32)		.01		1.52 (0.55-4.22)		.42		1.19 (0.67-2.13)			.55	
	Non-Hispanic	—		—		—		—		—			—	
**Race**														
	Asian	0.84 (0.48-1.46)		.53		0.86 (0.16-4.68)		.86		1.21 (0.63-2.34)			.56	
	Black or African American	1.01 (0.70-1.47)		.96		1.56 (0.66-3.70)		.31		1.05 (0.62-1.77)			.86	
	Other race^d^	0.56 (0.33-0.95)		.06		0.44 (0.10-2.03)		.29		0.49 (0.21-1.14)			.10	
	White (reference)	—		—		—		—		—			—	
Comfort with technology			1.05 (0.95-1.16)		.36		1.44 (1.02-2.06)		.04		1.00 (0.87-1.15)			.99
**Baseline PHQ-8^e^**														
	Moderate	1.25 (0.84-1.85)		.27		4.88 (1.37-17.34)		.01		0.77 (0.46-1.29)			.32	
	Moderately severe	1.46 (0.99-2.17)		.06		5.34 (1.47-19.38)		.01		0.85 (0.51-1.43)			.54	
	Severe	1.06 (0.65-1.72)		.81		3.10 (0.67-14.33)		.15		0.59 (0.29-1.19)			.14	
	Mild (reference)	—		—		—		—		—			—	

^a^ODB: online discussion board.

^b^OR: odds ratio.

^c^Not available (indicates reference value).

^d^Other race: the other race category constitutes those identified as Pacific Islander, Native American, mixed, or other.

^e^PHQ-8: Patient Health Questionnaire-8.

Participants rated the facilitator phone calls as moderately enjoyable (mean 47.20, SD 32.64; range 0-100), and the qualitative feedback supported a wide range of responses such as “I liked hearing from a real person, because then I knew someone was paying attention to what I was doing in the course” versus “It got irritating to answer the same questions and it was frustrating and embarrassing because I hadn't done the skills.” Enjoyment ratings of FC were significantly correlated with staff contact by phone (Spearman *r*_107_=0.27; *P=*.006) and the FC enjoyment ratings were positively associated with the number of skills accessed (Spearman *r*_98_=0.25; *P=.*01).

#### Analysis of the ODB

Among participants assigned to receive the ODB enhancement (n=262), 25.2% (66/262) posted on the discussion board at least once, and slightly less than half 48.5% (127/262) liked or commented on a post at least once. Male participants posted a greater number of times on the ODB than female participants (*P*=.02). The number of times that participants posted on the ODB was positively correlated with the number of skills accessed (Spearman *r*_198_=0.49; *P<*.001).

Enjoyment ratings for the ODB were also in the midrange (mean 45.53, SD 30.69; range 0-100), although the ratings varied greatly. Some participants appreciated connecting with other participants through the discussion board: “It was motivational to see others progress and be able to communicate with others.” However, for others, the ODB fell short of expectations: “There doesn’t seem to be much activity there.” Still others were in the middle ground and neither liked or disliked it: “I was able to connect with other people in the program but it wasn’t very personal, in the sense that I would just comment a few blurbs here and there but didn’t feel like I was doing much.” Those who rated the ODB as more enjoyable had more posts on the board (Spearman *r*_101_=0.29; *P=.*003). However, participants’ ODB enjoyment ratings were not associated with the number of skills accessed in the program (Spearman *r*_91_=0.10; *P=.*34).

#### Analysis of VB

Among participants who were assigned to receive a VB enhancement (n=260), the number of badges earned ranged from 9 to 192, with a mean of 17.29 (SD 16.48). All participants assigned to receive a VB enhancement earned at least one badge. As seen in Table 3, older participants and Hispanic participants earned a greater number of VB than younger and non-Hispanic participants (*P*=.01 to *P*=.05). None of the other baseline characteristics significantly predicted the total number of VB (all *P*=.06 to *P*=.96).

For enjoyment ratings, participants rated the VB as moderately enjoyable (mean 50.90, SD 33.17; range 0-100). However, there was wide variability in enjoyment ratings, and the qualitative feedback for the VB reflected this range: “It is a visual point system that is cute and fun. It seems silly but I felt like I accomplished something when I got each flower.” Others did not appreciate the whimsical garden plot calling it *childish* and *a waste of time*. Although the number of badges earned and enjoyment ratings of the VB enhancement were significantly correlated (Spearman *r*_115_=.30; *P=*.001), participants’ VB enjoyment ratings did not predict the number of skills accessed (Spearman *r*_109_=0.14; *P=.*16).

## Discussion

### Principal Findings

Web-based, self-guided interventions hold significant promise for people with elevated depressive symptoms. Attaining acceptable levels of adherence to these programs is critical to effectiveness, yet this has proven to be a challenge. We developed and tested 3 enhancements that we hypothesized would, either alone or in combination, improve adherence to a self-guided positive psychological intervention for people with elevated depression. The enhancements were an ODB, VB, and FC. Participants in the eight conditions that received the intervention content also received 1, 2, or all 3 or no enhancements. Our results suggest that the combination of VB and FC is especially impactful and points to areas for future focus to improve adherence across the three enhancements.

For the ODB enhancement, participants had access to an online forum where they could anonymously post questions, share their experiences, and offer encouragement to other participants. Previous findings regarding the efficacy of discussion boards for improving adherence to web-based programs have been mixed. For example, one study found that an internet discussion board or support group resulted in *lower* adherence to the self-guided program compared with the self-guided program alone [47], although participants who were assigned to receive the discussion board had a greater reduction in depression at 6 months.

The engagement on the discussion board was quite low. Only a quarter of the participants posted on the board at least once, and qualitative feedback indicated that this low level of activity further discouraged participants from making use of the ODB. Therefore, it is perhaps not surprising that there was no indication that the ODB increased adherence to the program, either alone or in combination with other enhancements. Simple availability without engagement is not sufficient for an ODB to increase adherence to the intervention content. There are a number of ways that we could have increased engagement in the ODB, such as requiring participants to post as part of the home practice assignments or making it easier to navigate to the discussion board to lower the barriers to engagement.

VB is a form of gamification and is intended to increase adherence to the intervention content by providing rewards for completing activities on the platform, for example, reaching a goal for the number of log-ins or consecutive homework completions. Participants in the arm that received both VB and FC completed significantly more sessions than did those in the no-enhancement arm (Figure 2). Furthermore, participants in arms that received VB and FC together (VB+FC; VB+FC+ODB) accessed, on average, one more skill (mean 6.42) compared with participants who received either VB or FC without the other (mean 5.42; FC alone, VB alone, ODB+VB, and ODB+FC). Participants were of a mixed opinion on whether the VB were enjoyable, with 33.9% (39/115) giving the VB low enjoyment ratings (below 31 on a 0-100 scale) and 33% (38/115) providing high enjoyment ratings (between 70 and 100). Previous studies document similar challenges to the successful use of gamification elements, such as a lack of clarity on which behaviors are being rewarded or confusion over how progress was being illustrated [48-50]. Future work to make the badges more interpretable, enjoyable, and clearly linked to target behaviors will help to increase the impact of the VB on adherence to the intervention.

Our FC enhancement consisted of staff contacting participants by phone once per week for a brief check-in regarding any challenges they were experiencing while completing that week’s skills and answering questions or concerns they had. We included the FC component based on studies showing that contact with a person improves adherence to self-guided, web-based interventions [18,23,27,51,52]. Although receipt of the FC alone did not significantly improve adherence to the program, when combined with VB, there was an improvement such that those participants who received *both* FC and VB completed more of the intervention sessions compared with those who did not. The average number of times a facilitator was able to reach a participant was one, and fewer than half of the participants were ever reached by a facilitator by phone. The data indicate that the more participants enjoyed the FC, the more skills they completed, and as with VB, there was a wide range of enjoyment ratings for FC, leaving a lot of room for improvement. One easy change that may increase participants’ enjoyment of the FC condition, and as a result improve the impact of FC on adherence, is to vary the questions that are asked each week and word them so they do not come across as judgmental. Qualitative feedback indicated that participants found these questions repetitive and, especially if they had not completed the home practice that week, somewhat shaming. Furthermore, some participants may prefer email over phone contact, and providing an email contact option could increase the impact of the enhancement. Other ways to improve the impact of the FC condition could include improving the *supportive accountability* [29], such as setting clear expectations and goals for engagement with the platform, and tailored performance monitoring that is supportive rather than shaming. In addition, clarifying the expertise of the facilitators and increasing trust could enhance the impact of FC [29].

Aside from the content of the enhancement, a simple count of the number of enhancements the participant was assigned to (up to 3) was not predictive of adherence. On the basis of our data, it is not necessarily better. However, the combination of VB and FC increased adherence compared with either enhancement alone. It may be that the FC helps encourage people to engage with the platform, with the phone call providing a reminder and the facilitator providing someone to be accountable. Once participants are on the platform, the VB reinforce that engagement and provide some extrinsic motivation to remain engaged [29].

We explored potential moderators of the effect of each enhancement and found that depression influenced whether VB enhancement was associated with adherence. Specifically, among those with moderately severe baseline depression (PHQ-8 scores between 15 and 19), those who received the VB enhancement demonstrated better adherence than those who were not assigned to a VB condition. For those in the mild and moderate range of the PHQ-8 (scores between 5 and 14), the direction of the effect was the same but did not reach statistical significance. For participants with severe baseline depression (>20), the effect was in the opposite direction, such that VB were associated with (nonsignificantly) poorer adherence. Although this interaction should be interpreted with caution given the marginal significance and the very small number of participants in this group (those with the highest levels of depression who received the VB), this pattern was also apparent in our analyses with the proportion of intervention completed as an outcome (the number of pages viewed as a proportion of the total possible number of pages; Multimedia Appendix 1). It may be that for people with severe clinical depression, the badges were off-putting, and extra thought should be put into this form of gamification for people with the highest levels of depression. Of note, other studies of web-based depression interventions tend to exclude people who have scores in the severe range [10,20] so would not have picked up this potential demotivating effect of VB for people with the highest levels of depression.

### Limitations

There are several limitations to consider in this study. First, the sample was recruited from all web-based sources and likely differed from clinic-based samples or those referred by medical providers. The sample was predominantly made up of non-Hispanic White individuals and females, which significantly limits the generalizability of the findings. Furthermore, participants needed to complete 4 of 7 daily emotion reports as part of a *run-in* to be assigned to a condition in which only 58.05% (602/1037) of the participants were able to accomplish. Although run-in periods are often used in clinical trials [53], by including only those who demonstrated adherence to the study protocol, we may have artificially enriched our sample with participants who were more likely to be adherent to the intervention. Comparison of those who completed the run-in to those who did not indicate that those who completed were older, more likely to be female, and less depressed than those who consented but did not complete the run-in. We also ran a large number of statistical tests and did not control for multiple comparisons. However, our analyses were hypothesis-driven and appropriate for this stage of intervention development. Finally, we tested the enhancements in the context of a positive psychological intervention, and the content may have influenced adherence, although we do not have a way to test this possibility in this study.

These findings lead to several suggestions for future self-guided interventions and potential adherence enhancements. First, the program and enhancements were feasible even for people with very high levels of depression. Unlike most previous studies of web-based programs for people with depression, we included participants with the highest levels of depression. The findings demonstrate that people with severe levels of depression can also engage in programs like this, and their inclusion in the study sample suggested important differences in terms of response to enhancements. Second, the combination of VB and FC was associated with better adherence than either enhancement alone. Researchers should go beyond single enhancements to improve adherence and consider including combinations of enhancements that may be more effective than any one enhancement alone. Furthermore, it may be helpful to offer participants a choice of which enhancements they want to access. Providing participant control over which enhancements they receive from the outset could increase feelings of engagement and investment in the program. Finally, as the technology for intervention delivery progresses, there are new possibilities for better-engaging participants. Future work may consider more immersive approaches such as virtual reality [54] and approaches such as SMS text messages [55] or chatbots [56] that foster a sense of social connection and may encourage stronger engagement with the intervention content.

Self-guided web-based interventions hold great promise to help people living with depression, and creative approaches to better engage participants will provide greater benefit to more people as the programs are more widely disseminated. Despite low levels of engagement with the FC enhancement, the results of our randomized trial suggest that the combination of VB and FC may be especially effective at improving adherence to the intervention, which in turn, may increase the impact of the intervention on well-being and bode well for future programs that incorporate enhancements such as these.

## Appendix

Multimedia Appendix 1 Supplemental analyses.

Multimedia Appendix 2 CONSORT-eHEALTH checklist (V 1.6.1).

## Abbreviations

CONSORT: Consolidated Standards of Reporting Trials
FC: facilitator contact
ODB: online discussion board
PHQ-8: Patient Health Questionnaire-8
VB: virtual badges
