# Supplementary material for: Facilitator Contact, Discussion Boards, and Virtual Badges as Adherence Enhancements to a Web-Based, Self-guided, Positive Psychological Intervention for Depression: Randomized Controlled Trial
Source: J Med Internet Res. 2021 Sep 22;23(9):e25922. doi: 10.2196/25922 (PMC8495567; doi:10.2196/25922)
Supplement: Multimedia Appendix 1 [file jmir_v23i9e25922_app1.docx]

**Multimedia Appendix**

1. **Comparisons of Participants who Completed the Run-In Period vs. those who Did Not:**

See Supplemental Table 1 for the analyses comparing participants who completed at least 4 of the 7 days of the run-in period (and thus were randomized into one of the 9 study groups) with those who did not. Compared to those who did not complete at least 4 of the 7 days of the run-in period, participants who completed the run-in were older (*M* = 37.95 vs. 36.03, *p* = .04), more likely to be female (74% vs. 65%, *p* = .008), and had lower baseline depressive symptom severity (PHQ-8: *M* = 13.85 vs. 15.34, *p* < .001).

1. **Study Retention**

See Supplemental Table 2 for the analyses comparing study retention (% retained at each follow-up assessment) across the 9 study conditions (8 intervention conditions and 1 emotion reporting control condition). There were no differences in retention for any follow-up assessment as a function of condition, all *p*s > ,17.

We conducted a binary logistic regression within participants in the 8 intervention conditions predicting likelihood of retention (e.g., 1 = retained, 0 = not retained) at the post assessment by the number of enhancements (0 to 3) received. The total number of enhancements that participants received (0 to 3) did not predict study retention, χ^2^ = 1.94, *p* = .16.

**Study retention as a function of enhancement.** We conducted additional analyses within participants in the 8 intervention condition predicting study retention as a function of whether the participant received each enhancement and their two- and three-way interactions. None of the main effects, two-way or three-way interactions emerged in statistical significance, all *p*s > .24.

**Baseline predictors of retention.** We examined age, comfort with technology, race, ethnicity, gender, and baseline depression as predictors of study retention at post. As seen in Supplemental Table 3, Black/African American participants were more likely to be retained in the intervention at the post assessment relative to White participants*, p* = .05. Furthermore, participants who began the study with moderately severe levels of depression were less likely to be retained in the intervention at the post assessment relative to participants who began the study with mild levels of depression, *p* = .007.

1. **Days of Emotion Reporting Completed:**

See Supplemental Table 2 for the analyses comparing the number of days of emotion reporting completed across the 9 study conditions (8 intervention conditions and 1 emotion reporting control condition). We found that the number of days of daily emotion reporting that participants completed differed as a function of study condition, *F*(593) = 5.19, *p* < .001. Specifically, we found that participants in the emotion reporting control arm completed more days of daily emotion reports relative to participants in the following 4 (of 8) intervention arms: Int + FC, Int + VB, Int + FC + ODB, and Int + ODB + VB (all *p*s < .01). Participants in the emotion reporting control arm did not differ from those in the intervention only arm and the Int + ODB, Int + FC + VB, or Int + FC + ODB + VB arms in the number of daily emotion reports completed (*p*s > .13). In addition, participants in the Int + ODB arm completed more days of daily emotion reports relative to participants in the Int + FC + ODB arm, *p* = .03. None of the other intervention arms differed from one another on the number of days of daily emotion reporting completed, all *p*s > .07,

1. **Additional Measures of Adherence:**

Although the results in the main manuscript focused on the number of skills accessed as the primary indicator of participant adherence, we also examined three additional measures of adherence: 1) proportion of intervention content completed (the number of pages viewed as a proportion of the total possible number of pages, as there are several pages for each skill), 2) the number of skills participants practiced in the home work (8 total), and 3) the total number of days that participants completed their daily home practice (out of 42 total days). The pattern of findings for these additional adherence measures parallel the findings of the number of skills practiced, reported in the main text of the manuscript. We report the findings from these additional measures here:

**Adherence Outcome #1: Proportion of Intervention Content Completed**

We first conducted a linear regression examining whether the total number of enhancements received predicted the proportion of intervention content completed. Then we conducted the analyses predicting the proportion of intervention content completed from intervention arm, with the intervention only condition as the reference category. The total number of enhancements that participants received (0 to 3) did not predict the proportion of the intervention that participants completed, B = 0.07, *t*(420) = 1.50, *p* = .14.

**Proportion of intervention content complete as a function of enhancement.** We conducted additional analyses predicting the number of skills that participants practiced as a function of whether the participant received each enhancement and their two- and three-way interactions. We found a significant FC × VB two-way interaction on the number of skills practiced *F*(1, 414) = 6.31, *p* = .01. Simple effects tests revealed that participants in arms that received the combination of both the VB and FC enhancements together practiced a greater number of skills (*M* = 0.76, *SE* = 0.03) relative to participants who received either enhancement without the other: VB without FC (*M* = 0.65, *SE* = 0.03), *p* = .02, and FC without VB (*M* = 0.59, *SE* = 0.03), *p* < .001. Furthermore, participants who received either VB or FC enhancement without the other (*M* = 0.65, *SE* = 0.03) and (FC + no VB; *M* = 0.59, *SE* = 0.03) did not differ in the number of skills practiced compared with participants who received neither enhancement (*M* = 0.64, *SE* = 0.03), all *ps* > .25.

**Baseline predictors and moderators of the proportion of intervention content completed**

We examined age, comfort with technology, race, ethnicity, gender, and baseline depression as predictors of adherence and as moderators of effects of each individual enhancement on adherence. As seen in Supplemental Table 3, none of these baseline characteristics significantly predicted the proportion of intervention content completed, *p*s ≥ .19.

We then examined age, comfort with technology, race, ethnicity, gender, and baseline depression as moderators of the effect of each individual enhancement on the proportion of intervention content completed. Age, comfort with technology, race, ethnicity, and gender did not significantly moderate the effects of enhancement (FC, ODB, and VB) on the proportion of intervention content completed. However, baseline depressive symptom severity did appear to moderate the effects of VB of the proportion of intervention completed.

Baseline depressive symptom severity moderated the effects of the VB enhancement on the proportion of the intervention completed, as indicated by a significant baseline depressive symptom severity × VB two-way interaction, *F*(3, 406) = 3.76, *p* = .01. When examining the effects of receiving virtual badges for each of the four levels of depressive symptom severity separately, we found that receiving the virtual badge enhancement (vs. not receiving the virtual badge enhancement) was associated with completing a higher proportion of the intervention for those who had baseline depressive symptom severity in the mild (B = 0.16, *p* = .01), moderate (B = 0.11, *p* = .06), and the moderately severe ranges (B = 0.14, *p* = .02) (PHQ-8 scores < 20), although the effect for moderate depressive symptom severity was only marginal in statistical significance. However, for participants who had baseline depressive symptom severity in the severe range (PHQ-8 scores ≥ 20), the virtual badge enhancement was negatively associated with the proportion of the intervention completed, B = -0.16, *p* = .05. Baseline depressive symptom severity did not moderate the ODB or the FC enhancements on the proportion of the intervention content completed.

**Adherence Outcome #2: Number of Skills Practiced**

We conducted an overdispersed Poisson regression examining whether the number of enhancements (0 to 3) received predicted the number of skills practiced. Then we conducted the analyses predicting skills practiced from intervention arm, with the intervention only condition as the reference category. The total number of enhancements that participants received (0 to 3) did not predict the number of skills that participants practiced, χ^2^ = 1.75, *p* = .19.

**Number of skills practiced as a function of enhancement.** We conducted additional analyses predicting the number of skills that participants practiced as a function of whether the participant received each enhancement and their two- and three-way interactions. We found a significant FC × VB two-way interaction on the number of skills practiced χ^2^ = 11.24, *p* = .001. Simple effects tests revealed that participants in arms that received the combination of both the VB and FC enhancements together practiced a greater number of skills (*M* = 4.67, *SE* = 0.19) relative to participants who received either enhancement without the other: VB without FC (*M* = 3.85, *SE* = 0.17), *p* = .001, and FC without VB (*M* = 3.67, *SE* = 0.16), *p* = .001. Furthermore, participants who received either VB or FC enhancement without the other (VB + no FC: *M* = 3.85, *SE* = 0.17) and (FC + no VB; *M* = 3.67, *SE* = 0.16) did not differ in the number of skills practiced compared with participants who received neither enhancement (*M* = 3.91, *SE* = 0.17), all *ps* > .14.

None of the other two-way or three-way interactions emerged in statistical significance, all *p*s > .11. Taken together, these findings suggest that receiving both the VB and FC enhancements in combination increases the number of skills that participants practice, but receiving either the VB or FC enhancement without the other did not.

**Baseline predictors and moderators of the number of skills practiced**

We examined age, comfort with technology, race, ethnicity, gender, and baseline depression as predictors of adherence and as moderators of effects of the number of skills practiced. As seen in Supplemental Table 3, female participants and Hispanic participants practiced a greater number of skills than male and non-Hispanic participants respectively, *p*s < .02.

We then examined age, comfort with technology, race, ethnicity, gender, and baseline depression as moderators of the effect of each individual enhancement on the number of skills practiced. Age, race, ethnicity, and comfort with technology did not significantly moderate the effects of enhancement (FC, ODB, and VB) on the number of skills practiced. However, gender, baseline depressive symptom severity, and race did appear to moderate the effects of enhancements.

**Gender and ODB.** We found a significant gender × ODB two-way interaction in predicting the number of skills practiced, χ^2^ = 4.51, *p* = .03. Simple slopes analyses revealed that men who received the ODB enhancement practiced *fewer* skills relative to men who did not receive the ODB enhancement, (B = -1.19, *p* = .03). In contrast, there was no effect of receiving the ODB enhancement on home practice for women, *p* = .70.

**Baseline depressive symptom severity and VB.**  We found a significant baseline depressive symptom severity × VB two-way interaction for the number of skills practiced, χ^2^ = 7.87, *p* = .05. When examining the effects of receiving virtual badges for each of the four levels of depressive symptom severity separately, we found that receiving virtual badges (vs. not receiving virtual badges) was positively associated with the number of skills practiced for those who had baseline depressive symptom severity in the mild range (B = 1.16, *p* = .05) but not for those with symptoms in the moderate, moderately severe, or the severe range, all *p*s > .11.

**Race and VB.**  We found that the effects of the VB enhancement on the number of skills practiced differed as a function of race (Asian vs. Black/African vs. Other Race (Pacific Islander, Native American, Mixed/Other) vs. White (reference)). Specifically, we found a significant Other Race × VB two-way interaction for the number of skills practiced, χ^2^ = 9.22, *p* = .002. Simple effects tests revealed that among participants in the Other Race category (Pacific Islander, Native American, Mixed/Other), receiving virtual badges was negatively associated with the number of skills practiced (B = -2.17, *p* = .02). In contrast, among participants in the White category, there was no association between virtual badges and the number of skills practiced completed, *p* = .43.

**Adherence Outcome #3: Days of Home Practice Completed (out of 42 total).**

We conducted an overdispersed Poisson regression examining whether the number of enhancements (0 to 3) received predicted the number of days participants completed their daily home practice. The total number of enhancements that participants received (0 to 3) did not predict the number of days participants completed their daily home practice, χ^2^ = 0.79, *p* = .38.

**Days completed home practice as a function of enhancement.** We found a significant FC × ODB two-way interaction on the total number of days participants completed home practice, χ^2^ = 53.07, *p* < .001. Simple effects tests revealed that participants in arms that received the FC enhancement without ODB completed their home practice on a greater number of days (FC + no ODB: *M* = 10.16, *SE* = 0.28) relative to participants who received the combination of both the FC and ODB enhancements together (FC+ODB: *M* = 7.10, *SE* = 0.24), *p* < .001, and relative to participants who received neither enhancement (no FC + no ODB: *M* = 8.49, *SE* = 0.24), *p* < .001. Participants who received the ODB enhancement without FC (ODB + no FC: *M* = 9.13, *SE* = 0.26) did not differ from participants who received neither enhancement on the number of days of home practice completed (no FC + no ODB: *M* = 8.49, *SE* = 0.25), *p* = .08.

We also found a significant VB × ODB two-way interaction on the total number of days participants completed home practice, χ^2^ = 88.61, *p* < .001. Simple effects tests revealed that participants in arms that received the VB enhancement without ODB completed a greater number of days of home practice (VB + no ODB: *M* = 11.70, *SE* = 0.29) relative to participants who received the combination of both the VB and ODB enhancements together (VB +ODB: *M* = 7.68, *SE* = 0.25), *p* < .001, and relative to participants who received neither enhancement (no VB + no ODB: *M* = 7.37, *SE* = 0.23), *p* = .002. Participants who received the ODB enhancement without VB (ODB + no VB: *M* = 8.45, *SE* = 0.25) completed a greater number of days of home practice relative to participants who received neither enhancement (no VB + no ODB: *M* = 7.37, *SE* = 0.23), *p* = .03.

None of the other two-way or three-way interactions emerged in statistical significance, all *p*s > .88. Taken together, these findings suggest that receiving the ODB enhancement in combination with either the FC or VB enhancement may reduce the number of days participants complete home practice.

**Baseline predictors and moderators of the number of skills practiced.** We examined age, comfort with technology, race, ethnicity, gender, and baseline depression as predictors of adherence and as moderators of effects of the number of days participants completed home practice. As seen in Supplemental Table 3, older participants, female participants, and participants who were more comfortable with technology completed a greater number of home practice days than younger and male participants respectively, *p*s < .04.

We examined age, comfort with technology, race, ethnicity, gender, and baseline depression as moderators of the effect of each individual enhancement on the number of days participants completed home practice. Age, race, ethnicity, and comfort with technology did not significantly moderate the effects of enhancement (FC, ODB, and VB) on the number of days participants completed home practice. However, baseline depressive symptom severity and race did appear to moderate the effects of enhancements.

**Baseline depressive symptom severity and VB.**  We found a significant baseline depressive symptom severity × VB two-way interaction for the number of days participants completed home practice, χ^2^ = 8.47, *p* = .04. When examining the effects of receiving virtual badges for each of the four levels of depressive symptom severity separately, we found that receiving virtual badges was positively associated with the number of days participants completed home practice for those who had baseline depressive symptom severity in the mild range (B = 4.61, *p* = .075), though the effect was only marginally significant, but there was no association between virtual badges was associated with the number of days of home practice for those with symptoms in the moderate, moderately severe, or the severe range, all *p*s > .14.

**Race and VB.**  We found that the effects of the VB enhancement on the number of days participants completed home practice differed as a function of race (Asian vs. Black/African vs. Other Race (Pacific Islander, Native American, Mixed/Other) vs. White (reference)). Specifically, we found a significant Other Race × VB two-way interaction for the number of days participants completed home practice, χ^2^ = 10.56, *p* = .001. Simple effects tests revealed that among participants in the Other Race category (Pacific Islander, Native American, Mixed/Other), receiving virtual badges was negatively associated with the number of days of home practice completed (B = -8.95, *p* = .02). In contrast, among participants in the White category, there was no association between virtual badges and the number of days of home practice completed, *p* =.65.

Table S1. Comparison of Participants who Completed the Run-In Period (and thus were randomized into the study) vs. those who Did Not Complete the Run-In Period

|  | Did Not Complete Run-in (N=323) | Completed Run-in (N=633) | *p* |
| --- | --- | --- | --- |
| Age *M* (SD) | 36.03 (12.77) | 37.95 (13.68) | .04 |
| Gender (%) |  |  | .85 |
| Male | 34.4% | 25.3% | - |
| Female | 65.0% | 73.8% | - |
| Baseline PHQ-8 score *M* (SD) | 15.34 (5.11) | 13.85 (5.18) | <.001 |
| Ethnicity (%) |  |  | .82 |
| Hispanic | 17.3% | 16.7% | - |
| Not Hispanic | 82.7% | 83.3% | - |
| Race (%) |  |  |  |
| Black | 20.4% | 16.9% | .18 |
| White | 67.2% | 72.8% | .07 |
| Asian | 9.0% | 8.7% | .88 |
| Native American | 5.3% | 4.9% | .81 |
| Pacific Islander | 0.3% | 0.6% | .51 |
| Mixed/Other | 7.1% | 7.4% | .86 |

Table S2. Study Retention and Days of Emotion Reporting Completed by Condition.

|  | Total Sample | *Condition* | | | | | | | | | Group Difference  *p*-value |
| --- | --- | --- | --- | --- | --- | --- | --- | --- | --- | --- | --- |
|  |  | Int Only | Int + FC | Int + ODB | Int + VB | Int + FC + ODB | Int + FC + VB | Int + ODB + VB | Int + FC +ODB +VB | Emotion Reporting Control |  |
| *Retention* |  |  |  |  |  |  |  |  |  |  |  |
| Post N (%) | 311 (51.7%) | 36 (49.3%) | 33 (46.5%) | 37 (53.6%) | 34 (50.7%) | 26 (39.4%) | 39 (59.1%) | 31 (48.4%) | 41 (65.1%) | 34  (54.0%) | .17 |
| FU1 N (%) | 291 (48.3%) | 35 (47.9%) | 30 (42.3%) | 35 (50.7%) | 31 (46.3%) | 29 (43.9%) | 34 (51.5%) | 27 (42.2%) | 38 (60.3%) | 32  (50.8%) | .55 |
| FU2 N (%) | 302 (50.2%) | 36 (49.3%) | 32 (45.1%) | 38 (55.1%) | 31 (46.3%) | 32 (48.5%) | 36 (54.5%) | 27 (42.2%) | 39 (61.9%) | 31  (49.2%) | .48 |
| *Days of Emotion Reporting Completed* *M* (SD) | 12.23 (14.19) | 13.05 (13.54) | 10.55 (11.77) | 14.22 (15.34) | 8.85 (12.71) | 6.33 (10.90) | 13.68 (14.16) | 10.00 (12.03) | 13.86 (14.80) | 19.90 (18.01) | <.001 |

Table S3. Regressions Predicting Adherence and Retention from Baseline Predictors

|  | Proportion of Intervention Completed | | | Number of Skills Practiced (8 total) | | | Total Number of Days Participants Completed Home Practice | | | % Retained at Post | | |
| --- | --- | --- | --- | --- | --- | --- | --- | --- | --- | --- | --- | --- |
|  | B | *t* | *p* | OR | 95% CI | *p* | OR | 95% CI | *p* | OR | 95% CI | *p* |
| Age | 0.01 | 0.47 | .64 | 1.01 | 1.00-1.01 | .09 | **1.02** | **1.01-1.03** | **.001** | 1.01 | 1.00-1.02 | .17 |
| Gender |  |  |  |  |  |  |  |  |  |  |  |  |
| Female | 0.05 | 1.32 | .19 | **1.26** | **1.07-1.50** | **.007** | **1.44** | **1.01-2.05** | **.04** | 1.26 | 0.82-1.91 | .29 |
| Male (ref) | - | - | - | - | - | - | - | - | - | - | - | - |
| Ethnicity |  |  |  |  |  |  |  |  |  |  |  |  |
| Hispanic | -0.01 | -0.11 | .92 | **1.26** | **1.04-1.53** | **.02** | 1.26 | 0.84-1.89 | .26 | 1.22 | 0.72-2.07 | .47 |
| Non-Hispanic (ref) | - | - | - | - | - | - | - | - | - | - | - | - |
| Race |  |  |  |  |  |  |  |  |  |  |  |  |
| Asian | -0.03 | -0.47 | .64 | 0.88 | 0.68-1.15 | .36 | 0.75 | 0.41-1.38 | .36 | 0.90 | 0.47-1.70 | .74 |
| Black/African | 0.05 | 1.03 | .30 | 1.16 | 0.97-1.39 | .11 | 1.19 | 0.83-1.72 | .35 | **1.65** | **1.00-2.71** | **.05** |
| Other Race^a^ | 0.01 | 0.12 | .91 | 0.81 | 0.64-1.03 | .09 | 1.03 | 0.64-1.65 | .91 | 0.69 | 0.38-1.28 | .24 |
| White (ref) | - | - | - | - | - | - | - | - | - | - | - | - |
| Comfort with Technology | 0.01 | 0.98 | .33 | 1.03 | 0.98-1.09 | .25 | **1.17** | **1.04-1.33** | **.01** | 1.00 | 0.88-1.14 | .99 |
| Baseline PHQ-8 |  |  |  |  |  |  |  |  |  |  |  |  |
| Moderate | 0.06 | 1.27 | .20 | 1.06 | 0.88-1.28 | .56 | 1.13 | 0.77-1.64 | .53 | 0.84 | 0.51-1.38 | .49 |
| Moderately Severe | 0.02 | 0.56 | .65 | 0.94 | 0.77-1.14 | .51 | 1.08 | 0.73-1.60 | .70 | **0.50** | **0.30-0.82** | **.007** |
| Severe | 0.05 | 0.88 | .38 | 0.99 | 0.79-1.25 | .96 | 0.91 | 0.57-1.48 | .71 | 0.66 | 0.37-1.18 | .16 |
| Mild (ref) | - | - | - | - | - | - | - | - | - | - | - | - |
